# Mild cognitive impairment: when nutrition helps brain energy rescue—a report from the EuGMS 2020 Congress

**DOI:** 10.1007/s41999-021-00534-z

**Published:** 2021-07-05

**Authors:** Stephen C. Cunnane, Cornel C. Sieber, Russell H. Swerdlow, Alfonso J. Cruz-Jentoft

**Affiliations:** 1 Research Center on Aging and Department of Medicine, Université de Sherbrooke, Québec, Canada; 2 Department of Internal Medicine, Kantonsspital Winterthur, Winterthur, Switzerland; 3 University of Kansas Alzheimer’s Disease Center, KUMC Neurodegenerative Disorders Program, University of Kansas School of Medicine, Lawrence, KS USA; 4 Servicio de Geriatría, Hospital Universitario Ramón y Cajal (IRYCIS), Ctra. Colmenar km 9.1, Madrid, 28034 Spain

**Keywords:** Mild cognitive impairment, Nutrition, Medium-chain triglycerides

## Abstract

**Background:**

Mild cognitive impairment (MCI) is characterized by a decline in cognition and mainly affects older individuals above the age of 60. The global incidence of MCI varies, but it is often underdiagnosed and untreated. There is a distinct lack of approved pharmacologic options to treat MCI. There is, however, evidence to support the efficacy of nutritional interventions, such as ketogenic supplements/diets, which offer ketones as an alternative energy source to brain cells. This article explores the effect of ketones on metabolic activity in the brain and the mechanisms by which ketogenic medium-chain triglycerides (kMCTs) induce ketosis in patients with MCI.

**Key takeaways:**

This article reviews the effect of ketogenic supplements/diets on brain metabolism, including evidence supporting the efficacy of ketones as an efficient fuel for the brain. It discusses the use of oral nutritional ketogenic supplements, with particular reference to the 6-month randomized controlled BENEFIC trial, which showed that consumption of a kMCT drink, BrainXpert Energy Complex, improved cognitive performance in individuals with MCI compared with placebo.

**Conclusion:**

While there is a need for more long-term studies, results from the BENEFIC trial revealed the benefits of a brain-specific ketogenic supplement, as a nutritional intervention, on cognitive performance in individuals with MCI.

## Key Summary Points

**Aim:**

The prevalence of MCI is underestimated owing to underdiagnosis, resulting in a lack of timely intervention and undetected disease progression. This article aims to assess the effect of ketogenic supplements/diets on brain metabolism, including evidence supporting the efficcy of ketones as an efficient fuel for the brain.

**Findings:**

The 6-month randomized controlled BENEFIC trial showed that consumption of a ketogenic MCT drink improved cognitive performance in individuals with MCI compared with placebo.

**Message:**

There is evidence to support the efficacy of nutritional interventions such as ketogenic supplements/diets, which offer ketones as an alternative energy source for brain cells.

## Introduction

Mild cognitive impairment (MCI) can be defined as a transitional stage between normal aging and dementia [1]. It is seen as a decline in cognitive functioning that has gone beyond the expected level considering the patient‘s age and education [2], which does not yet impair activities of daily living [3]. The global MCI incidence is approximately 20 per 1000 person-years for individuals aged between 60 and 80 years [4–6]; however, this may still be an underestimation owing to underdiagnosis of the condition. Estimates for the incidence of MCI have broad ranges [4, 5, 7, 8] and variations within these estimates are mainly due to the diversity in sample populations (in terms of age and size) [5, 9], the constantly evolving definitions of MCI, the heterogeneity of diagnostic criteria, and the increasing incidence of MCI with age in general [4].

Variability in prevalence estimates also exist [10–13] for the same reasons identified for incidence estimate variability [5, 14, 15]. Additionally, ethnicity, urban/rural settings and varied study designs, including mixed sample sources (e.g. clinic-based versus community-based samples) [8, 10, 13, 16], make interpretation of results challenging. Accurately estimating the overall prevalence of MCI is made even more difficult as, according to one Cochrane review, 35% of MCI cases progress to dementia or Alzheimer’s disease (AD) over 5 years [17].

The World Health Organization considers the increasing incidence and prevalence of worldwide dementia a ‘public health priority’. By 2050, it is projected that there could be 115.4 million people living with dementia worldwide, contributing to a significant epidemiologic and economic burden to communities and countries. These projected global figures for dementia make treating MCI effectively and delaying its conversion to AD a matter of urgency and necessity [18].

**Fig. 1:**
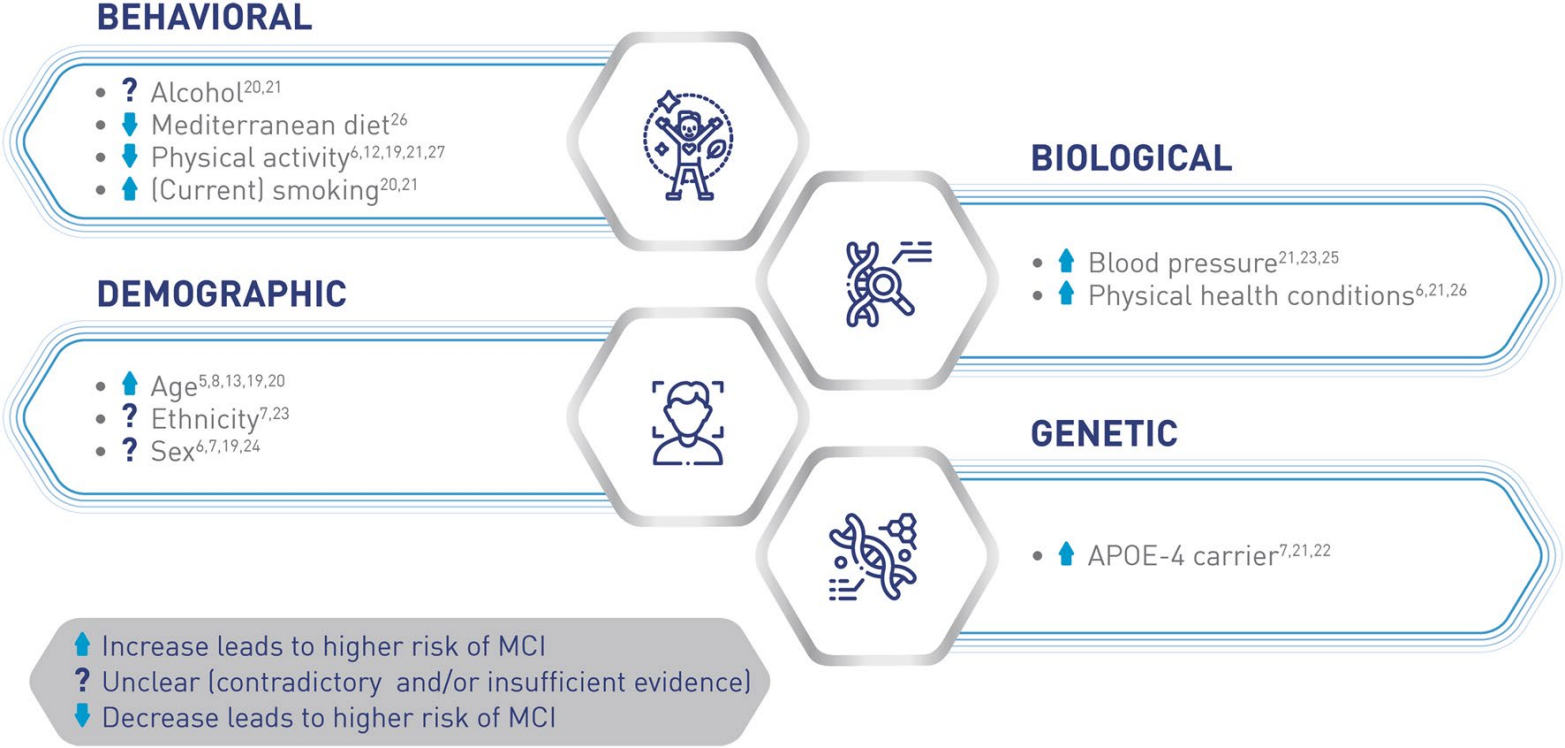
Risk factors for mild cognitive impairment. *APOE* apolipoprotein E, *MCI* mild cognitive impairment

Risk factors for MCI are also diverse (Fig. 1). Demographic factors such as older age [5, 8, 13, 19, 20] and being a genetic carrier of apolipoprotein E4[7, 21, 22] are non-modifiable risk factors associated with MCI. Ethnicity [7, 23] and sex [6, 7, 19, 24] are also thought to contribute to the risk of developing MC,I but evidence that exists is not sufficient to draw any firm associations. The presence of existing biological conditions, such as elevated blood pressure [21, 23, 25], Type 2 diabetes, and insulin resistance has also been associated with an increased risk of MCI [6, 21, 26]. There is some evidence to show that behavioral risk factors, such as alcohol consumption and smoking [20, 21], may be modifiable, and measures taken to reduce their frequency may slow the development of MCI. There are protective factors that have been associated with lowered risk of developing MCI, such as increased physical activity [6, 12, 19, 21, 27]. There is also epidemiological evidence to suggest that maintaining a Mediterranean diet is linked with reduced risk of developing MCI and AD, as well as a decreased risk of MCI progressing to AD [26].

As MCI is a transitional stage between normal aging and dementia and AD [1], it is important for clinicians and scientists to find ways to delay MCI disease progression [14]. Nutrition is a key modifiable determinant of healthy aging but there is a lack of published articles that address how cognitive improvement in MCI can be achieved through nutritional approaches. Optimal nutritional support is a crucial component of effective geriatric care, making this developing field one of great interest for geriatricians.

## Mild cognitive impairment: a silent and late-detected disease

### Underdiagnosis and misdiagnosis of mild cognitive impairment

There are validated screening tools for detecting MCI but underdiagnosis remains a challenge. While the mini-mental state examination (MMSE) is the most widely used instrument, the test is time consuming and its sensitivity for detecting MCI is low [28, 29]. The Montreal Cognitive Assessment (MoCA) [30] demonstrated 97% sensitivity for detecting MCI and MCI/AD combined but validating the MoCA using healthy controls may overestimate its specificity. While the MoCA may be suitable for assessing whether an individual does or does not need further diagnostic investigation, it may not be able to identify cognitive impairment [31]. Similarly, DemTect is sensitive but with low specificity [30]. The General Practitioner Assessment of Cognition (GPCOG) [32] is recommended for routine dementia screening in general practice as GPCOG has been validated in community, population, or general practice samples [30]. The challenge with many diagnostic tools is that these tools do not detect early stages of the disease or, once cognitive impairment is observed, development of MCI may already have progressed to early stages of dementia. For example, cerebrospinal fluid (CSF) biomarkers [33] are well established and cost effective as AD predictors but along with lumbar puncture, are only performed in memory clinics that do CSF sampling. In most cases, individuals referred to memory clinics for CSF sampling tend to be at a more advanced stage in the dementia continuum [29].

Although MCI is defined by a decline in cognitive function, non-cognitive neuropsychiatric symptoms can also occur and may be indicative of an increased risk of progression to dementia [28, 30]. Longitudinal assessments are, therefore, necessary for directly measuring cognitive change over time to track disease progression from normal cognition to MCI. Amyloid-β biomarkers and neuropsychiatric symptoms are measurable even before the development of MCI, meaning that patients may be identified prior to the onset of cognitive decline, which offers the potential for earlier intervention [34, 35].

### Progression from mild cognitive impairment to dementia

**Fig. 2:**
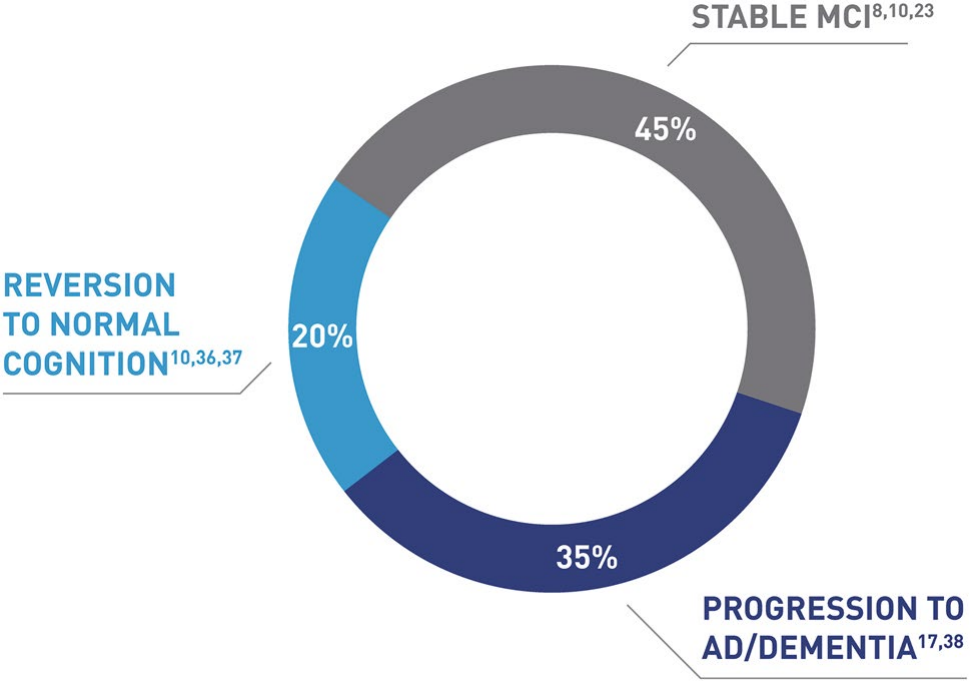
Progression from mild cognitive impairment to dementia. *AD* Alzheimer’s disease, *MCI* mild cognitive impairment

Sometimes MCI can be reversible, but this is very much dependent on its cause [23]. Approximately 20% of people with MCI return to normal cognition [10, 36, 37] and 45% remain stable with MCI [8, 10, 23]. It is estimated that approximately 35% of people progress to AD or other forms of dementia during a time period of around 5 years (Fig. 2) [17, 38]. As the pathological process begins years before the onset of AD, it is widely agreed that early identification of MCI is of great importance [39]. Individuals with MCI demonstrate objective cognitive impairment and report subjective complaints, but have relatively intact functional abilities [40, 41]. Accurate identification of early stages of dementia in individuals is vital for initiating therapeutic interventions.

### Improving cognitive function: the role of nutrition

Early diagnosis of cognitive decline would allow early therapeutic intervention, with the aim of delaying progression to dementia and AD. However, currently, drug treatment studies have not demonstrated sustainable results for MCI [19]. Despite years of clinical trials, no pharmacologic treatments have been approved by drug regulatory agencies for either the treatment of MCI or for delaying the development of MCI into dementia [19, 42]. Although the 2018 guidelines from the American Academy of Neurology for MCI state that there are currently no approved nutritional interventions for MCI [19], studies are increasingly demonstrating the potential of nutritional interventions in improving MCI [43, 44]. Ketogenic medium-chain triglycerides (kMCTs) are able to provide ketones as an alternative energy source to the brain, and thereby compensate for deteriorating brain glucose uptake during aging [45–47] and AD [46, 48].

### Effect of ketones on metabolic activity in the brain

The 2018 update of the American Academy of Neurology guideline on MCI recommends that clinicians advise patients with MCI that there are currently no “pharmacologic or dietary agents shown to have symptomatic cognitive benefit in MCI and that no medications have been approved for this purpose”. These recommendations emphasize the need for new therapeutic approaches for managing and treating MCI [19]. As a result, understanding the underlying mechanisms of MCTs on brain activity has become an area of great interest.

A recently published review found that patients with MCI experience approximately 10% decrease in their usual brain glucose metabolism resulting in a chronic brain energy shortage or brain energy gap [45]. The brain requires a sustained supply of energy, which predominantly comes in the form of glucose from oxidative phosphorylation in the mitochondria [45]. While brain glucose uptake is compromised in MCI, brain ketone uptake and metabolism remain normal in both MCI and mild-moderate AD [45]. Hence, ketones produced in the liver can act as alternative energy substrates for the brain [45].

Ketogenic diets are high-fat, very low-carbohydrate diets that mimic the metabolic profile of fasting and permit the body to produce ketones endogenously [49, 50]. kMCT supplements generate ketones independent of the macronutrient profile of the diet, thereby providing an alternative energy source for brain cells [50].

Ketogenic interventions, either through fasting or kMCT administration, have been shown to impact the brain and its function. Plasma ketones normally contribute to 2–5% of the brain’s energy requirements, but when available in moderately increased amounts, they are preferentially taken up by the brain over glucose [51]. Proposed neuroketotherapeutic mechanisms are thought to include the bioenergetic effects of ketones and their ability to support oxidative phosphorylation. Ketones reportedly uncouple respiration, affecting histone acetylation through which they also impact gene expression [49]. Ketones also appear to act as substrates at receptors, where they can affect sympathetic nervous system dysfunction and reduce inflammation [49].

Ketotherapeutics for MCI and AD have been investigated in a number of preliminary studies [52, 53]. Studies report that consuming a ketogenic diet improved memory in individuals with MCI [54] and demonstrated enhanced cognitive scores in participants with AD [55, 56] when compared with those on a placebo diet. While initial data demonstrate a neurocognitive benefit in individuals experiencing cognitive decline, more investigations are required in larger sample populations [54]. There is mounting evidence to suggest that endogenous and exogenous sources of ketones may partially bypass brain glucose hypometabolism and improve brain energy metabolism in both MCI and mild-moderate AD [46, 51, 57, 58].

### Oral nutritional supplements for mild cognitive impairment (BENEFIC trial)

**Fig. 3:**
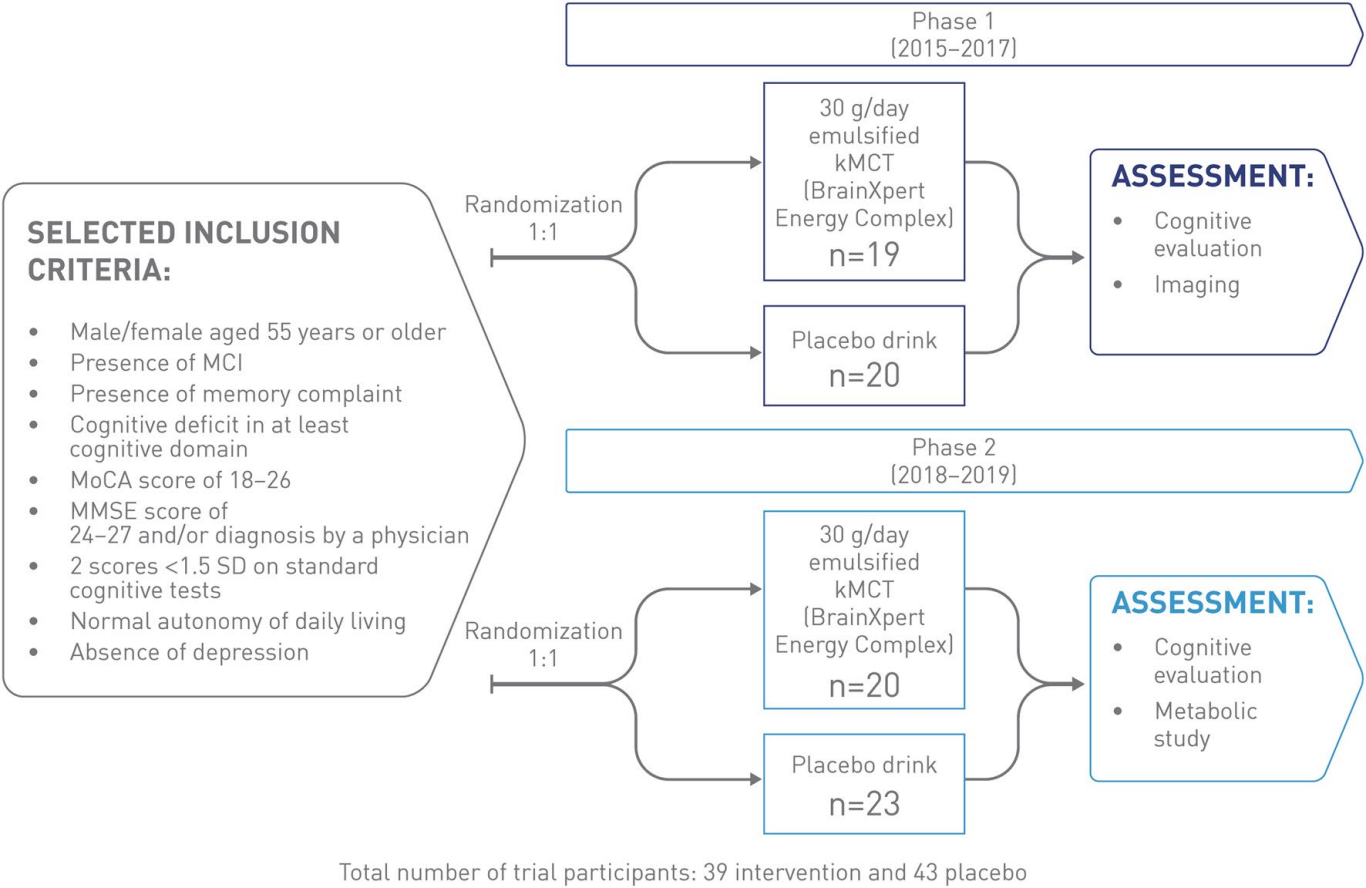
BENEFIC trial design [51, 58]. *kMCT* ketogenic medium-chain triglyceride, *MCI* mild cognitive impairment, *MMSE* mini-mental state examination, *MoCA* Montreal Cognitive Assessment, *SD* standard deviation

The Brain ENErgy, Functional Imaging, and Cognition (BENEFIC) randomized controlled trial was conducted in two phases to assess improvements in cognitive performance in MCI and AD (Fig. 3). Phases 1 and 2 combined established that a kMCT drink, BrainXpert Energy Complex, improved cognition in MCI [51, 58].

**Fig. 4:**
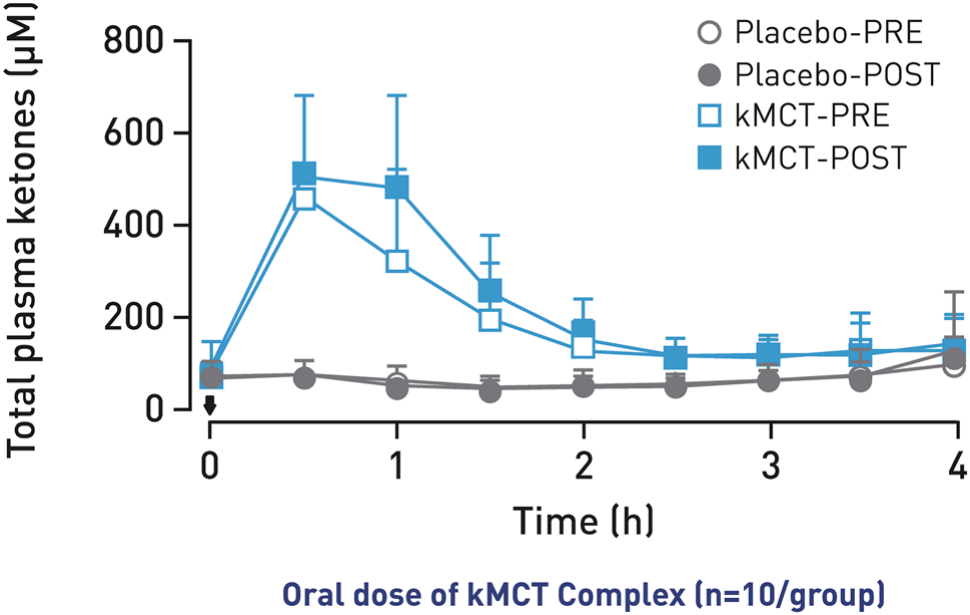
Sustained blood ketone response throughout the 4-h metabolic trial day. Arrow depicts when first dose of 15 g of kMCT drink or placebo was consumed. kMCT, ketogenic medium-chain triglyceride. Figure adapted with permission from Fortier et al. [58]

A total of 39 participants who received kMCT and 43 who received placebo completed both phases [51]. Men and women aged 55 and over were recruited for the two phases. Included individuals had MCI (Peterson criteria, 2004) [59], the presence of a subjective memory complaint, objective cognitive deficit in at least one cognitive domain, a MoCA score of 18–26, an MMSE score of 24–27 and/or diagnosis by a physician, two scores <1.5 standard deviations on standard cognitive tests, normal autonomy of daily living, and an absence of depression. Phase 1 demonstrated that consumption of kMCT, a drink providing 30 g/day of emulsified kMCT, enhanced brain energy in participants with MCI compared with a calorie-matched long-chain fatty-acid placebo. Positron emission topography imaging showed increased brain ketone uptake and plasma measurements showed sustained blood ketone response in individuals with MCI after 6 months of consuming kMCT (Fig. 4). The second phase of the BENEFIC trial involved a larger overall sample size to enable statistical differences in cognitive effects between interventional and placebo groups to be observed [51, 58].

**Fig. 5:**
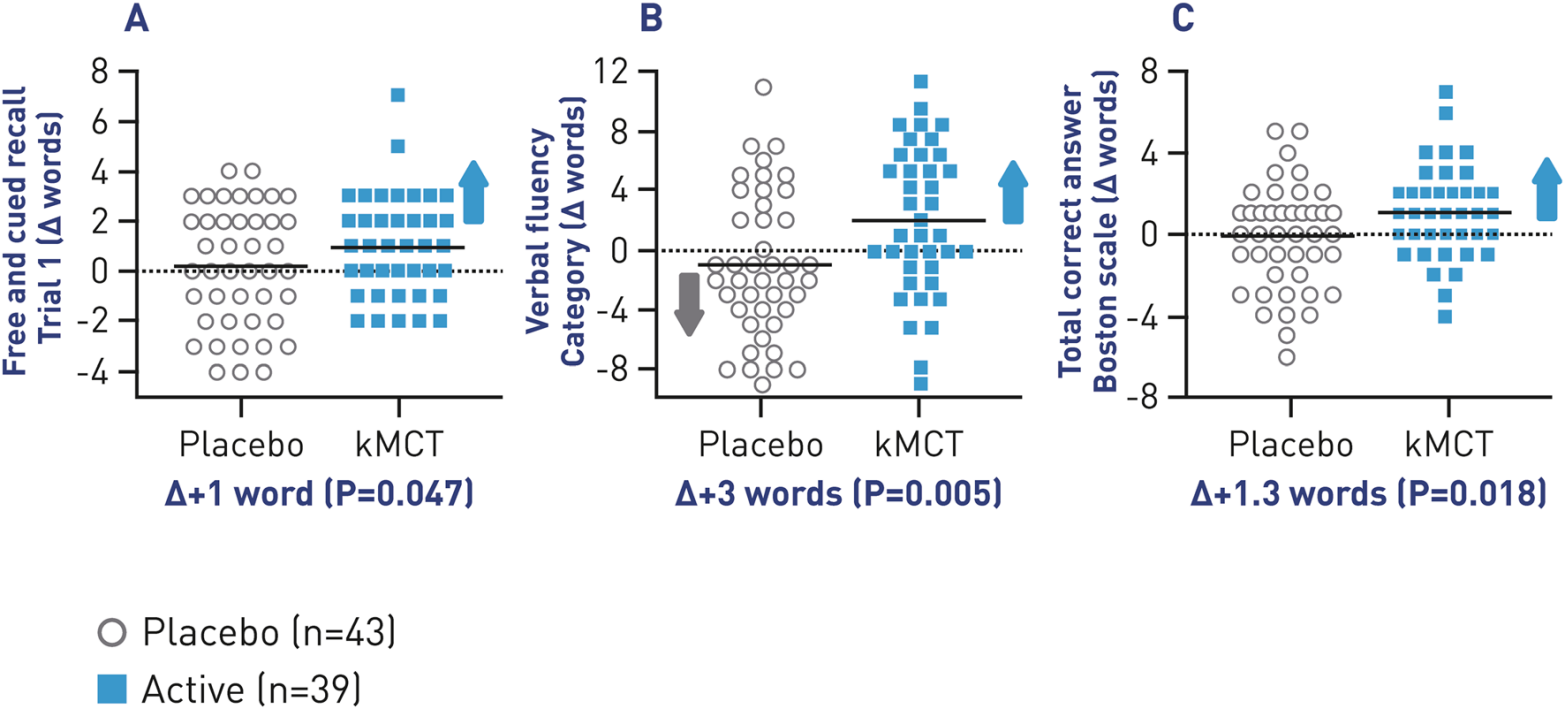
Change in cognitive scores. Change in raw scores from baseline (0) on the first trial of **A** Free and cued recall test (*P* = 0.047); **B** Verbal fluency (*P* = 0.005); **C** Boston Naming Test (*P* = 0.018). *kMCT* ketogenic medium-chain triglyceride. Figure has been adapted with permission from Fortier et al. [58]

The BENEFIC trial found that participants with MCI who consumed BrainXpert Energy Complex demonstrated improved raw scores on several cognitive tests—the free and cued recall test (*P* = 0.047), verbal fluency (categories; *P* = 0.005), and the Boston Naming test (*P* = 0.018) (Fig. 5). The differences between the kMCT and placebo groups were statistically significant. Higher plasma or brain ketone levels also correlated positively with the cognitive changes observed on these four cognitive tests, showing that the improved brain energy status was at least in part responsible for the improved cognitive results. Overall, the trial established that BrainXpert Energy Complex was well tolerated and feasible for an MCI population to consume twice daily for 6 months. Blood lipids were also reported—total cholesterol increased by 6% in the kMCT group but remained within the clinical reference range (4.9–5.2 mM).[58]These results provide robust support for the concept that brain energy rescue with ketones can improve cognitive outcomes in both MCI and AD. While further investigations are required to determine the optimal dosage for maximum cognitive benefit of kMCTs, the results of the BENEFIC trial hold promise for future therapeutic treatment with BrainXpert Energy Complex for MCI.

## Conclusions

MCI is part of the dementia continuum and therefore, early identification of the pathological progress as well as longitudinal assessments for tracking disease progression are essential. Ketogenic interventions provide an important alternative energy supply when brain glucose stores are compromised. While existing ketogenic diet studies establish ketotherapeutic potential in the treatment of MCI, reported trials are currently exploratory and have mostly assessed feasibility; further investigation is therefore warranted. The randomized controlled BENEFIC trial demonstrated that BrainXpert Energy Complex improved certain cognitive outcomes in MCI in direct relation to the net change in brain energy status. Further investigation with a larger sample size will now be required to determine the long-term sustainability of the cognitive improvement. Overall, however, studies such as BENEFIC show the feasibility and potential of long-term clinical trials with kMCTs in older people.

## Data Availability

Not applicable.

## References

[1] Petersen RC, Smith GE, Waring SC, Ivnik RJ, Tangalos EG, Kokmen E (1999). Mild cognitive impairment: clinical characterization and outcome. Arch Neurol.

[2] Petersen RC, Doody R, Kurz A (2001). Current concepts in mild cognitive impairment. Arch Neurol.

[3] Association A (2019). 2019 Alzheimer's disease facts and figures. Alzheimers Dement.

[4] Gillis C, Mirzaei F, Potashman M, Ikram MA, Maserejian N (2019). The incidence of mild cognitive impairment: a systematic review and data synthesis. Alzheimers Dement (Amst).

[5] Ward A, Arrighi HM, Michels S, Cedarbaum JM (2012). Mild cognitive impairment: disparity of incidence and prevalence estimates. Alzheimers Dement.

[6] Mielke MM, Vemuri P, Rocca WA (2014). Clinical epidemiology of Alzheimer's disease: assessing sex and gender differences. Clin Epidemiol.

[7] Luck T, Luppa M, Briel S, Riedel-Heller SG (2010). Incidence of mild cognitive impairment: a systematic review. Dement Geriatr Cogn Disord.

[8] Roberts R, Knopman DS (2013). Classification and epidemiology of MCI. Clin Geriatr Med.

[9] Luis CA, Loewenstein DA, Acevedo A, Barker WW, Duara R (2003). Mild cognitive impairment: directions for future research. Neurology.

[10] Hu C, Yu D, Sun X, Zhang M, Wang L, Qin H (2017). The prevalence and progression of mild cognitive impairment among clinic and community populations: a systematic review and meta-analysis. Int Psychogeriatr.

[11] Petersen RC (2016). Mild cognitive impairment. Continuum (Minneap Minn).

[12] Vancampfort D, Stubbs B, Lara E, Vandenbulcke M, Swinnen N, Koyanagi A (2017). Mild cognitive impairment and physical activity in the general population: findings from six low- and middle-income countries. Exp Gerontol.

[13] Xue J, Li J, Liang J, Chen S (2018). The prevalence of mild cognitive impairment in China: a systematic review. Aging Dis.

[14] Anderson ND (2019). State of the science on mild cognitive impairment (MCI). CNS Spectr.

[15] Pioggiosi PP, Berardi D, Ferrari B, Quartesan R, De Ronchi D (2006). Occurrence of cognitive impairment after age 90: MCI and other broadly used concepts. Brain Res Bull.

[16] Alexander M, Perera G, Ford L (2015). Age-stratified prevalence of mild cognitive impairment and dementia in European populations: a systematic review. J Alzheimers Dis.

[17] Arevalo-Rodriguez I, Smailagic N, Roque IFM (2015). Mini-Mental State Examination (MMSE) for the detection of Alzheimer's disease and other dementias in people with mild cognitive impairment (MCI). Cochrane Database Syst Rev.

[18] World Health Organization and Alzheimer's Disease International (2012) Dementia: a public health priority, p 112

[19] Petersen RC, Lopez O, Armstrong MJ (2018). Practice guideline update summary: mild cognitive impairment: report of the guideline development, dissemination, and implementation subcommittee of the American Academy of Neurology. Neurology.

[20] Koyanagi A, Lara E, Stubbs B (2018). chronic physical conditions, multimorbidity, and mild cognitive impairment in low- and middle-income countries. J Am Geriatr Soc.

[21] Apostolo J, Holland C, O'Connell MD (2016). Mild cognitive decline. A position statement of the Cognitive Decline Group of the European Innovation Partnership for Active and Healthy Ageing (EIPAHA). Maturitas.

[22] Jefferson AL, Beiser AS, Seshadri S, Wolf PA, Au R (2015). APOE and mild cognitive impairment: the Framingham Heart Study. Age Ageing.

[23] Manly JJ, Tang MX, Schupf N, Stern Y, Vonsattel JP, Mayeux R (2008). Frequency and course of mild cognitive impairment in a multiethnic community. Ann Neurol.

[24] Au B, Dale-McGrath S, Tierney MC (2017). Sex differences in the prevalence and incidence of mild cognitive impairment: a meta-analysis. Ageing Res Rev.

[25] Cheng Y, Xiao S (2014). Recent research about mild cognitive impairment in China. Shanghai Arch Psychiatry.

[26] Singh B, Parsaik AK, Mielke MM (2014). Association of mediterranean diet with mild cognitive impairment and Alzheimer's disease: a systematic review and meta-analysis. J Alzheimers Dis.

[27] Kobayashi Y, Takahashi Y, Seki T (2016). Decreased physical activity associated with executive dysfunction correlates with cognitive impairment among older adults in the community: a retrospective analysis from the Kurihara project. Dement Geriatr Cogn Dis Extra.

[28] Ismail Z, Elbayoumi H, Fischer CE (2017). Prevalence of depression in patients with mild cognitive impairment: a systematic review and meta-analysis. JAMA Psychiat.

[29] Panegyres PK, Berry R, Burchell J (2016). Early dementia screening. Diagnostics (Basel)..

[30] Velayudhan L, Ryu SH, Raczek M (2014). Review of brief cognitive tests for patients with suspected dementia. Int Psychogeriatr.

[31] Dautzenberg G, Lijmer J, Beekman A (2021). Clinical value of the Montreal Cognitive Assessment (MoCA) in patients suspected of cognitive impairment in old age psychiatry. Using the MoCA for triaging to a memory clinic. Cogn Neuropsychiatry.

[32] Brodaty H, Low LF, Gibson L, Burns K (2006). What is the best dementia screening instrument for general practitioners to use?. Am J Geriatr Psychiatry.

[33] Teipel S, Kilimann I, Thyrian JR, Kloppel S, Hoffmann W (2018). Potential role of neuroimaging markers for early diagnosis of dementia in primary care. Curr Alzheimer Res.

[34] Ismail Z, Smith EE, Geda Y (2016). Neuropsychiatric symptoms as early manifestations of emergent dementia: provisional diagnostic criteria for mild behavioral impairment. Alzheimers Dement.

[35] Donovan NJ, Amariglio RE, Zoller AS (2014). Subjective cognitive concerns and neuropsychiatric predictors of progression to the early clinical stages of Alzheimer disease. Am J Geriatr Psychiatry.

[36] Canevelli M, Grande G, Lacorte E (2016). Spontaneous reversion of mild cognitive impairment to normal cognition: a systematic review of literature and meta-analysis. J Am Med Dir Assoc.

[37] Malek-Ahmadi M (2016). Reversion from mild cognitive impairment to normal cognition: a meta-analysis. Alzheimer Dis Assoc Disord.

[38] Han JW, Kim TH, Lee SB (2012). Predictive validity and diagnostic stability of mild cognitive impairment subtypes. Alzheimers Dement.

[39] Elman JA, Jak AJ, Panizzon MS (2018). Underdiagnosis of mild cognitive impairment: a consequence of ignoring practice effects. Alzheimers Dement (Amst).

[40] Edmonds EC, McDonald CR, Marshall A (2019). Early versus late MCI: improved MCI staging using a neuropsychological approach. Alzheimers Dement.

[41] Edmonds EC, Delano-Wood L, Clark LR (2015). Susceptibility of the conventional criteria for mild cognitive impairment to false-positive diagnostic errors. Alzheimers Dement.

[42] Farhang M, Miranda-Castillo C, Rubio M, Furtado G (2019). Impact of mind-body interventions in older adults with mild cognitive impairment: a systematic review. Int Psychogeriatr.

[43] Raschetti R, Albanese E, Vanacore N, Maggini M (2007). Cholinesterase inhibitors in mild cognitive impairment: a systematic review of randomised trials. PLoS Med.

[44] Chen X, Maguire B, Brodaty H, O'Leary F (2019). Dietary patterns and cognitive health in older adults: a systematic review. J Alzheimers Dis.

[45] Cunnane SC, Trushina E, Morland C (2020). Brain energy rescue: an emerging therapeutic concept for neurodegenerative disorders of ageing. Nat Rev Drug Discov.

[46] Cunnane SC, Courchesne-Loyer A, St-Pierre V (2016). Can ketones compensate for deteriorating brain glucose uptake during aging? Implications for the risk and treatment of Alzheimer's disease. Ann N Y Acad Sci.

[47] Jensen NJ, Wodschow HZ, Nilsson M, Rungby J (2020). Effects of ketone bodies on brain metabolism and function in neurodegenerative diseases. Int J Mol Sci.

[48] Avgerinos KI, Egan JM, Mattson MP, Kapogiannis D (2020). Medium Chain Triglycerides induce mild ketosis and may improve cognition in Alzheimer's disease. A systematic review and meta-analysis of human studies. Ageing Res Rev.

[49] Swerdlow RH, Koppel S, Weidling I, Hayley C, Ji Y, Wilkins HM (2017). Mitochondria, cybrids, aging, and alzheimer's disease. Prog Mol Biol Transl Sci.

[50] Augustin K, Khabbush A, Williams S (2018). Mechanisms of action for the medium-chain triglyceride ketogenic diet in neurological and metabolic disorders. Lancet Neurol.

[51] Fortier M, Castellano CA, Croteau E (2019). A ketogenic drink improves brain energy and some measures of cognition in mild cognitive impairment. Alzheimers Dement.

[52] Taylor MK, Sullivan DK, Mahnken JD, Burns JM, Swerdlow RH (2018). Feasibility and efficacy data from a ketogenic diet intervention in Alzheimer's disease. Alzheimers Dement (N Y).

[53] Neth BJ, Mintz A, Whitlow C (2020). Modified ketogenic diet is associated with improved cerebrospinal fluid biomarker profile, cerebral perfusion, and cerebral ketone body uptake in older adults at risk for Alzheimer's disease: a pilot study. Neurobiol Aging.

[54] Krikorian R, Shidler MD, Dangelo K, Couch SC, Benoit SC, Clegg DJ (2012). Dietary ketosis enhances memory in mild cognitive impairment. Neurobiol Aging.

[55] Henderson ST, Vogel JL, Barr LJ, Garvin F, Jones JJ, Costantini LC (2009). Study of the ketogenic agent AC-1202 in mild to moderate Alzheimer's disease: a randomized, double-blind, placebo-controlled, multicenter trial. Nutr Metab (Lond).

[56] Brandt J, Buchholz A, Henry-Barron B, Vizthum D, Avramopoulos D, Cervenka MC (2019). Preliminary report on the feasibility and efficacy of the modified Atkins diet for treatment of mild cognitive impairment and early Alzheimer's disease. J Alzheimers Dis.

[57] Vandenberghe C, St-Pierre V, Fortier M, Castellano CA, Cuenoud B, Cunnane SC (2020). Medium chain triglycerides modulate the ketogenic effect of a metabolic switch. Front Nutr.

[58] Fortier M, Castellano CA, St-Pierre V (2020). A ketogenic drink improves cognition in mild cognitive impairment: results of a 6-month RCT. Alzheimers Dement.

[59] Petersen RC (2004). Mild cognitive impairment as a diagnostic entity. J Intern Med.

